# Whole genome characterization of sequence diversity of 15,220 Icelanders

**DOI:** 10.1038/sdata.2017.115

**Published:** 2017-09-21

**Authors:** Hákon Jónsson, Patrick Sulem, Birte Kehr, Snaedis Kristmundsdottir, Florian Zink, Eirikur Hjartarson, Marteinn T. Hardarson, Kristjan E. Hjorleifsson, Hannes P. Eggertsson, Sigurjon Axel Gudjonsson, Lucas D. Ward, Gudny A. Arnadottir, Einar A. Helgason, Hannes Helgason, Arnaldur Gylfason, Adalbjorg Jonasdottir, Aslaug Jonasdottir, Thorunn Rafnar, Soren Besenbacher, Michael L. Frigge, Simon N. Stacey, Olafur Th. Magnusson, Unnur Thorsteinsdottir, Gisli Masson, Augustine Kong, Bjarni V. Halldorsson, Agnar Helgason, Daniel F. Gudbjartsson, Kari Stefansson

**Affiliations:** 1 deCODE genetics/Amgen Inc., Sturlugata 8, Reykjavik 101, Iceland; 2 Bioinformatics Research Centre (BiRC), C.F.Møllers Allé 8, Aarhus University, Aarhus 8000 Aarhus C, Denmark; 3 Department of Clinical Medicine—Molekylær Medicinsk afdeling (MOMA), Palle Juul-Jensens Boulevard 99, Aarhus University Hospital, Aarhus 8200 Aarhus N, Denmark; 4 Faculty of Medicine, School of Health Sciences, University of Iceland, Reykjavik 101, Iceland; 5 School of Engineering and Natural Sciences, University of Iceland, Reykjavik 101, Iceland; 6 School of Science and Engineering, Reykjavik University, Reykjavik 101, Iceland; 7 Department of Anthropology, University of Iceland, Reykjavik 101, Iceland

**Keywords:** Rare variants, Genetic variation, DNA sequencing, Haplotypes

## Abstract

Understanding of sequence diversity is the cornerstone of analysis of genetic disorders, population genetics, and evolutionary biology. Here, we present an update of our sequencing set to 15,220 Icelanders who we sequenced to an average genome-wide coverage of 34X. We identified 39,020,168 autosomal variants passing GATK filters: 31,079,378 SNPs and 7,940,790 indels. Calling *de novo* mutations (DNMs) is a formidable challenge given the high false positive rate in sequencing datasets relative to the mutation rate. Here we addressed this issue by using segregation of alleles in three-generation families. Using this transmission assay, we controlled the false positive rate and identified 108,778 high quality DNMs. Furthermore, we used our extended family structure and read pair tracing of DNMs to a panel of phased SNPs, to determine the parent of origin of 42,961 DNMs.

## Background & Summary

Characterization of genetic diversity is of paramount importance to population genetics. Advances in sequencing technologies have been instrumental in the creation of whole-genome sequencing data sets on the population level^1–5^. Sequencing data augmented with chip-typed samples have proven a valuable approach in genome-wide association studies, by combining the low cost of chip typing with the comprehensive characterization of sequence diversity via whole-genome sequencing^1–3,5^. In contrast, the analysis of DNMs and other recent mutations requires the sequencing of families in which these recent mutations have occurred.

DNM detection is hampered by the fact that they are typically only observed in very few individuals (most often a single individual) and sequencing unrelated individuals is of limited value. Furthermore, analysis of singletons and DNMs is complicated since current sequencing technologies and calling algorithms identify a non-trivial number of false-positives^6^. The analysis of rare variants requires more extensive quality control than the analysis of common variants.

Families with well understood inheritance patterns provide useful tools for this purpose, such as monozygotic twin concordance and the transmission of DNM alleles to offspring of probands in three-generation families. These methods provide genome-wide validation assays with no ascertainment beyond the family relationships of the probands. A useful feature of the Icelandic population are comprehensive genealogical records going back to 1,600 (and in some instances back to 740). The deCODE genetics genealogical databases currently includes records of 819,410 Icelanders^7^.

Here, we have sequenced 12,803 Icelanders in addition to the previously described 2,417 (ref.^ 7^) to an average 34X coverage. This sequencing effort resulted in 39,020,168 autosomal variants passing GATK filters and for the entire variant set we were able to impute 37,127,995 variants into a set of 151,677 chip genotyped Icelanders.

We particularly focused on obtaining quality DNM calls. We calibrated our DNM calling with segregation of alleles in three-generation families, and subsequently validated our DNM calls with concordance among monozygotic twins. This resulted in data set of 108,778 high quality DNM calls, of which we were able to determine the parent of origin for 42,961 by observing their segregation in three-generation families and read tracing DNMS with phased and imputed variants.

## Methods

The following methods are an extension of those reported by Jónsson *et al.*^8^

### Chip genotyping and long range phasing

We performed chip genotyping on 47,555 additional individuals relative to the set presented by Gudbjartsson *et al.*^7^. We used the OmniExpress24 chip for the majority of the samples (33,710; Table 1). This update resulted in a set of 151,677 individuals with chip genotypes. Finally, we long range phased (LRP) the data set using the algorithm described by Kong *et al.*^9^, resulting in a panel of 151,677 individuals with LRP chip genotypes.

### Preparation of samples for whole-genome sequencing

DNA was derived from two sample types: buccal swab and whole blood. Three different sample preparation kits from Illumina were employed: TruSeq DNA (Method A), TruSeq Nano (Method B) and TruSeq PCR-Free (Method C). Samples were prepared for sequencing according to the manufacturers’ instructions (Illumina). In short, either 50 ng (Method B) or 1 μg (Methods A and C) of genomic DNA, isolated from either frozen blood samples or buccal swabs, was fragmented to a mean target size of 300–400 bp using a Covaris E220 instrument. End repair, generating blunt ended fragments was performed followed by size selection using different ratios of AMPure XP magnetic purification beads. 3′-Adenylation and ligation of indexed sequencing adaptors containing a T nucleotide overhang was performed, followed either by AMPure purification (Method C) alone or purification followed by PCR enrichment (10 cycles) using appropriate primers (Methods A and B). The quality and concentration of all sequencing libraries was assessed using either the Agilent 2,100 Bioanalyzer (12-samples) or the LabChip GX (96-samples) instrument from Perkin Elmer. Sequencing libraries were diluted and stored at −20 °C. Further quality control of sequencing libraries was done by multiplexing and pooling either 24 or 96 samples and sequencing each pool on an Illumina MiSeq instrument to assess optimal cluster densities, library insert size, duplication rates and library diversities. All steps in the workflow were monitored using an in-house laboratory information management system (LIMS) with barcode tracking of all samples and reagents.

### DNA whole genome sequencing

Sequencing libraries were hybridized to the surface of paired-end (PE) flowcells using the Illumina cBot. Paired-end sequencing-by-synthesis (SBS) was performed on Illumina sequencers, including GAII_x_, HiSeq 2,000/2,500 or HiSeq X instruments, respectively. Read lengths depended on the instrument and/or sequencing kit being employed and varied from 2×76 cycles to 2×150 cycles of incorporation and imaging. Real-time analysis involved conversion of image data to base-calling in real-time. The largest number of samples (approximately 12,000) were sequenced on HiSeq X instruments with read lengths of 2×150 cycles, using either v1 or v2 flowcells and sequencing chemistries, respectively (Table 2).

### Reference

The reference sequences used to map reads was the human genome assembly GRCh38, not including alternate assemblies (GCA_000001405.15_GRCh38_no_alt_analysis_set.fna) in addition to sequences determined to represent common contaminants in our sequencing pipeline. These sequences are the sequences of the bacteriophage PhiX (Enterobacteria_phage_phiX174_sensu_lato_uid14015/NC_001422.fna), the bacteria *Ralstonia Pickettii* (Ralstonia_pickettii_12D_uid58859) and two sequences from the human microbiome (Coprobacillus_D7_uid32495/NZ_EQ999972.fna, Coprobacillus_D7_uid32495/NZ_EQ999922.fna).

### Alignment of sequences

We aligned the raw sequences against the reference described in the previous section with BWA version 0.7.10 mem^10^. The sequences in the BAM files were realigned around indels with GenomeAnalysisTKLite/2.3.9 (ref. 11) using a public set of known indels and a set of indels previously discovered in the Icelandic data. We marked PCR duplicates with Picard tools 1.117.

### Filtering of BAM files

We organized the sequencing data of each individual into one BAM file per lane on the flowcell. Much of the sequencing data generated for each individual prior to the HiSeqX machines derived from multiple lanes. In contrast, the sequencing data generated for each individual from HiSeqX machines largely derives from single lanes. In the following, we will refer to BAM files created before and after the merging per individual as BM- and AM-BAM files, respectively.

We performed a pileup using samtools BM-BAM files at all sites where the sample is homozygous according to their chip genotypes. We combined these pilups for each sample and calculated a mismatch rate as the number of bases not matching the chip genotype divided by the number of chip typed bases. We exlucded BM-BAM files with mismatch rates above 2% from the analysis.

We merged BM-BAM files into single AM-BAM files based on unique individual and sample type source combinations. The total number of AM-BAM files, prior to the subsequent quality filtering was 17,326. For contamination estimation, we used number and fraction of read pairs between pairs of heterzygote SNP markers supporting three or more haplotypes as a surrogate for the contamination level. We omitted AM-BAM files from subsequent analysis where more than 0.1% of SNP pairs had two or more reads supporting the third haplotype.

We removed AM-BAM files from the analysis if any of the underlying BM-BAM files failed any of the following criteria:

- Mean base quality <25
- Percent marked duplicate >50
- Mean N per read >30
- Percent mapping quality below 20 >11
- Percent reads unmapped >40
- Percent both reads in a pair unmapped >40
- Percent first read in a pair unmapped >40
- Percent second read in a pair unmapped >40

Furthermore, if there were two sample types available for the individual AM-BAM file, we used the whole-blood AM-BAM file.

The final set of AM- and BM-BAM files used for the genotyping by sequencing consisted of 15,220 and 27,817 files, respectively. The set of AM-BAM files derive from 14,101 blood and 1,119 buccal swab samples. The co-occurrences of flowcell and machine types for BM-BAM files are listed in Table 3. The counts of machine and flowcell combinations for the AM-BAM files are listed in Table 4.

### Variant calling

We applied a filtering step after merging the alignments and before the variant calling. We required the alignment to contain at least 45 matching bases (not necessarily consecutive) and removed parts of reads extending from template and into the adapter. For the adapter removal, we hard clip bases from the forward read extending further than the rightmost alignment of the reverse read and vice versa for the bases in the reverse read extending further than the leftmost alignment in the forward read.

Subsequently, we merge BAM files across all individuals in segments of 50,000 bases using SAMtools (version 1.3)^12^. These merged BAM files were used as input to GATK unified genotype caller^11^, resulting in a pooled genotype calling of 15,220 individuals. This resulted in 39,020,168 autosomal variants that passed the GATK recommended filters^1^, of which 31,079,378 and 7,940,790 were SNPs and indels, respectively (Table 5). Of the 15,220 individuals used for the sequence genotyping, we restricted the DNM identification to 14,688 individuals with over 20x coverage.

### Variant phasing

To improve genotype quality and to phase genotypes, we used an iterative algorithm based on the IMPUTE HMM model^13^ and LRP haplotypes^1,7^. This method is based on the principle that individuals that share haplotypes spanning the marker of interest are more likely to carry the same allele at the marker on the background of the shared haplotypes. For example, a pair of carriers of a rare sequence variant that share a haplotype are more likely to carry the rare variant on the shared haplotype than the two haplotypes that they do not share.

### Variant imputation

Variants were imputed based on the IMPUTE HMM model^13^ as previously described^1,7^, where chip genotyped individuals that share haplotypes with individuals in the set of sequenced individuals (the training set) are imputed to have the alleles on the background of the shared haplotypes.

### Genotype imputation information

The informativeness of genotype imputation was estimated by the ratio of the variance of imputed expected allele counts and the variance of the actual allele counts:

$$\frac{\mathrm{Var}(E\left(\theta |\mathrm{chipdata}\right))}{\mathrm{Var}(\theta )},$$

where *θ*∈{0, 1} is the allele count. Var(*E*(*θ*/chip data)) was estimated by the observed variance of the imputed expected counts and Var(*θ*) was estimated by *p*(1−*p*), where *p* is the allele frequency. The number of sequence variants with imputation information greater than 0.8 per variant type is listed in Table 5.

### Extraction of *de novo* candidates

**Figure 1:**
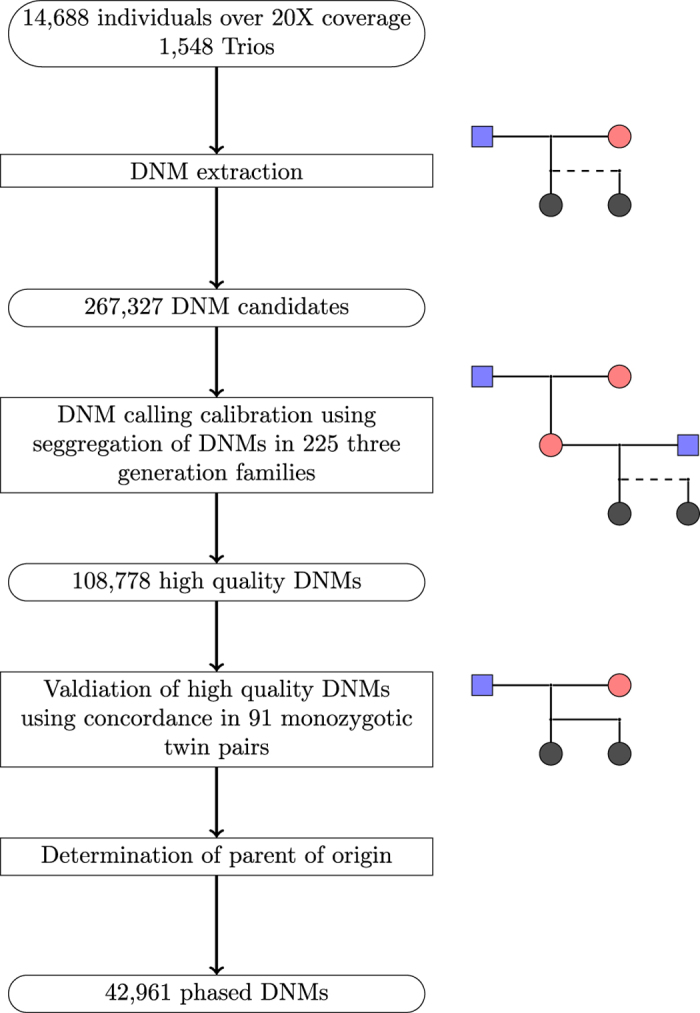
Schematic overview of the DNM characterization.

A schematic overview of the DNM identification procedure is shown in Fig. 1. Here we define allelic balance as the fraction of reads supporting the alternative allele out of the reads supporting the reference and alternative alleles combined. We used the genotype from GATK to define possible carriers. We define likely carriers of a DNM as those that meet the following requirements: read depth over 12, allele balance deviation from 0.5 less than 0.25 and genotype likelihood difference greater than 20 between the highest and second-highest scoring genotype (GQ). We restricted our DNM analysis to the primary assembly of the autosomes and chromosome X (hg38).

We extracted DNM candidates from the variants satisfying the following criteria:

- The proband must be an alternative allele carrier.
- For homozygous alternative allele carriers, we only consider candidates with 1 or no reads supporting the reference allele
- Minimum depth in the parent of 12 reads for the autosomes and 6 reads for the X chromosome in males
- No more than one read supporting the alternative allele in the parent
- Maximum allelic balance for parent of 0.05
- Minimum depth of 12 reads for proband
- Minimum allelic balance for proband of 0.15
- Maximum of 10 possible and 3 likely carriers beyond the descendants of the parent-pair
- Maximum of 10% average soft clipping per read covering the DNM candidate

Furthermore, we removed probands from the DNM analysis with more than: 10% average soft clipping per read, 1.5% average fraction of Ns in the read alignment and 300 DNM candidates. This removed 15 probands, of which two are three-generation probands and one was a monozygotic twin proband.

### QC-filtering using transmission in three-generation families

The DNM candidates identified in three-generation family probands were used to tune the DNM calling by tracing the transmission of alleles to offspring of the proband. We interpret discrepancy between haplotype sharing and segregation of a DNM as an indication that it is not present in the germline. More specifically, we dichotomized the DNMs according to whether they are consistent with haplotype transmission from the proband to his or her offspring. We restricted to DNMs where two or more offspring share distinct haplotypes from the proband in three-generation families.

We imposed a biological constraint on the DNM candidates on the X chromosome, i.e., male carriers must be homozygous for the alternative allele, otherwise we consider it inconsistent. To avoid inconsistency calls due to low quality genotypes in the offspring, we treated genotype calls of the offspring as missing if they do not meet the following requirements: at least 2 reads supporting an alternative allele, allelic balance over or equal to 0.1 and depth over or equal to 10 reads.

This evaluation of the DNM candidates gave us a binary outcome y, that we incorporate into the following Generalized Additive Model (GAM)

$$Y∼s(\mathrm{Psroband}{}_{-}\mathrm{allelic}{}_{-}\mathrm{balance})+s(\mathrm{oxoG}{}_{-}\mathrm{FoxoG}{}_{-}\mathrm{metric})\;+s(\mathrm{Trio}{}_{-}\mathrm{NPOSS})+\mathrm{gatk}{}_{-}\mathrm{filter}.$$

*s*() denotes a smooth term and the covariates are described in the following listing:

- Proband_allelic_balance, allelic balance of the proband.
- oxoG_FoxoG_metric, 0 for non-C>A substitutions and for C>A substitutions it measures the strand specific affinity of the C>A variant, as defined by Costello *et al.*^14^.
- Trio_NPOSS, The number of possible carriers of the DNM allele beyond the descendants of the proband’s parents. For example, if there are 11 possible carriers of the variant unrelated to the proband Trio_NPOSS is 11.
- gatk_filter, this is a binary covariate dichotomizing whether the variant passes variant filters recommended by GATK best practices.

**Figure 2:**
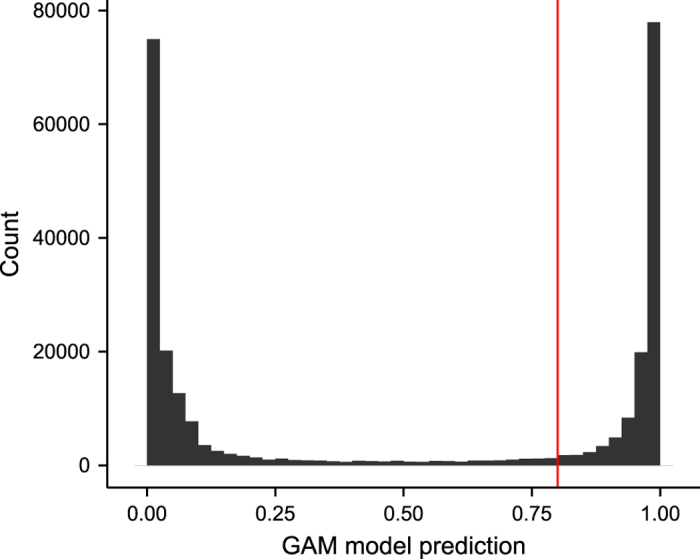
The GAM model predicted response for all DNM candidates. The red line corresponds to the 0.8 GAM response requirement for the high quality DNMs.

Using the fitted model, we predicted response for all DNM candidates. We used response strictly greater than 0.8 to define high-quality DNMs (Fig. 2).

### Phasing of *de novo* mutations

We used two complementary approaches to phasing DNMs, i.e., using haplotype sharing in three-generation families and read pair tracing DNMs with phased variants. In the former approach, we determined the parent of origin as in our previous analysis^15^. For example, if an offspring of the proband is a carrier of the DNM and carries the paternal chromosome of the proband, we assigned the mutation to the father. Conversely, if the offspring is not a DNM allele carrier then we would assign the DNM to the mother. If there were conflicting phase assignments among multiple siblings, we treated the DNM as unphased. We restricted the haplotype sharing analysis to segments of genetic length of at least 0.8 cM and with 200 consecutive markers present in the LRP panel.

In the latter approach, we used the phased and imputed panel of markers as described above to serve as a reference panel. We counted the number of read pairs traversing each biallelic combination between the DNM variant and the neighboring imputed and phased variants. We also recorded the number of read pairs supporting three or more haplotypes and reads not consistent with any of the biallelic combinations. We aggregated these numbers of read pairs per parent pair and DNM site. Using this summary, we phased DNMs by assigning the DNM allele to the parental chromosome with the read support. We only considered DNMs with read support for only one parent-of-origin. Finally, we aggregated the phase information from both methods into a consensus phase. Of the 4,566 DNMs that were phased by both methods, we excluded 53 that were phased inconsistently (Table 6).

### Code availability

We used publicly available software (URLs listed below) in conjunction with the above described algorithms in the sequencing processing pipeline (sections Variant phasing and Variant imputation).

- BWA 0.7.10 mem, https://github.com/lh3/bwa
- GenomeAnalysisTKLite 2.3.9, https://github.com/broadgsa/gatk/ https://github.com/broadgsa/gatk-protected/
- Picard tools 1.117, https://broadinstitute.github.io/picard/
- SAMtools 1.3, http://samtools.github.io/
- Bedtools v2.25.0-76-g5e7c696z, https://github.com/arq5x/bedtools2/

## Data Records

We have deposited two data records to the European Variation Archive (EVA), the 39,020,168 sequence variants passing the GATK filters in 15,220 sequenced Icelanders (Data Citation 1) and the 108,778 high quality DNMs (Data Citation 2) annotated with phase information.

## Technical Validation

As we have previously validated the accuracy of our imputation pipeline for rare and common variants^7^, we focused our validation efforts on the DNM set.

### Family relationship confirmation

There were reported 3,056 mother-child and 2,473 father-child connections among our 14,688 sequenced Icelanders (over 20X average coverage) as recorded in the genealogical database, of those we confirmed 2,981 and 2,386 based on haplotype sharing, respectively. This resulted in 1,548 trios and 225 three-generation families after restricting the analysis to probands passing the quality control.

### Three-generation set

We calibrated the cutoffs of our DNM calls via consistent segregation of alleles in three-generation families. We used consistent transmission as the dependent variable in a generalized additive model (GAM). We used the fitted GAM model to derive prediction for all the DNM candidates. A histogram of the prediction for all DNM candidates is shown in Fig. 2. There is a clean separation between variants that we predicted to have been transmitted to the next generation and those that are inconsistent with three-generation haplotype sharing.

### Concordance among monozygotic twins

**Figure 3:**
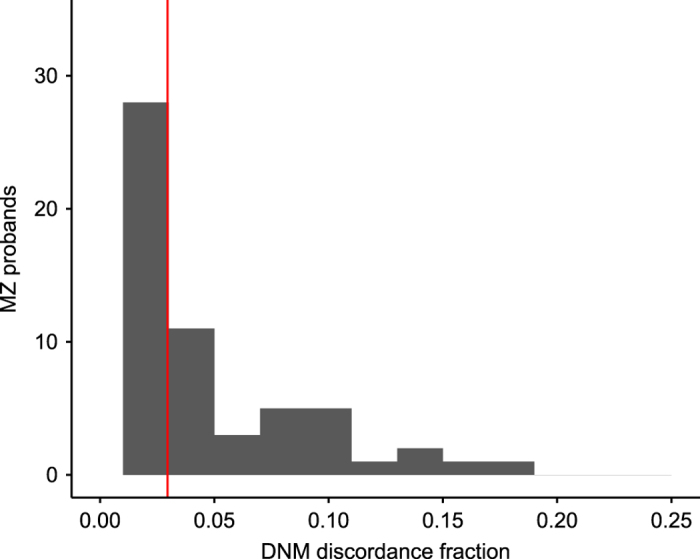
The fraction of discordant DNMs between MZ twins. There were used 91 monozygotic twin pairs for the discordance calculation. The discordance fraction was calculated as the fraction of the proband’s high quality DNMs not found in the MZ twin.

Only 8 of the 91 monozygotic twin probands were used for the DNM calling calibration. Thus, DNM concordance among the monozygotic twins serves as an independent validation metric of the DNM calls. We calculated the DNM discordance between each monozygotic twin proband and its monozygotic twin. We treated all genotype calls of the monozygotic twin as missing if the depth was less than 10 and heterozygous calls as missing if they did not meet the following requirements: at least 2 reads supporting an alternative allele and allelic balance over or equal to 0.1. We could verify the absence or presence of the genotype in the twin of the proband for 6,000 out of 6,034 DNMs and found low discordance (2.9%) for the high quality DNMs calls (Fig. 3). This shows that our DNM data set is of high quality genome-wide as there is no ascertainment of DNMs selected for validation beyond the monozygotic proband requirement.

### Phase comparison

We used two complementary approaches to assign parent of origin to the DNMs, the haplotype sharing between the parents and offspring of three-generation proband, and physically linking DNM candidates with phased variants via read pair tracing. We compared the concordance and discrepancy between the phasing methods. We found a low discrepancy 1.16% between the phasing approaches (Table 6).

## Additional Information

**How to cite this article:** Jónsson, H. *et al.* Whole genome characterization of sequence diversity of 15,220 Icelanders. *Sci. Data* 4:170115 doi: 10.1038/sdata.2017.115 (2017).

**Publisher’s note:** Springer Nature remains neutral with regard to jurisdictional claims in published maps and institutional affiliations.

## Supplementary Material



## Figures and Tables

**Table 1:** Summary of the genotyping chips used for the individuals in the LRP panel.

**Name**	**Description**	**Nr. of SNPs**	**Current**	**Overlap**
OmniExpress	HumanOmniExpress	725,095	49,482	35,847
OmniExpress24	HumanOmniExpress-24	714,758	33,894	184
HumanHap 300	Illumina HumanHap 317 K SNP Chip	317,870	22,429	22,394
HumanHapCNV 370	Merge of HumanHap300 and HumanCNV chips	371,900	14,138	14,085
Human Omni1	1 M SNP Chip redesigned, 500 K diff versus normal 1 M	1,137,466	10,859	10,809
OmniExpPlus	DECODE	706,534	9,725	9,708
Omni2.5–8	HumanOmni2.5–8	2,379,855	3,948	3,941
OmniExpMulti	HumanOmniExp-12v1MultiUse	730,525	2,808	2,799
HumanOmni2.5	HumanOmni2.5	2,443,177	2,352	2,340
HumanHap 1 M	Illumina HumanHap 1 M SNP Chip	1,136,004	1,267	1,261
Omni5	HumanOmni5-4v1	4,301,332	661	658
HumanHap 610	Illumina Human610-Quad v1 SNP Chip	620,901	646	640
Omni2.5Multi	HumanOmni2.5-4v1-Multi_D	2,443,177	400	399
Human660W	Human660W	655,214	22	1
The overlap column corresponds to the numbers of genotyping chips used for individuals that were present in the LRP set of Gudbjartsson *et al.*^1^ The Nr. of SNPs column is the average number of markers across all minor versions of the chip type.				

**Table 2:** The pairwise co-occurrences of sample preparation and flow-cell type.

	**HiSeq X**	**HiSeq X2**	**HiSeq 2000**	**HiSeq 2500**	**GAIIx**	**HiSeq**
TrueSeq DNA	6,603	452	107	0	0	0
TruSeq Nano	0	5,310	1,054	124	0	0
TruSeq PCR-Free	0	0	6	0	21	1
Unknown	0	0	4	1	0	0
This table is based on the subset of individuals sequenced and used for genotyping by sequencing in this study. Note that the same individual can appear multiple times if sequenced more than once. The sample preparation methods are described in the ‘Preparation of samples for whole genome sequencing’ section. This table is reproduced from Supplementary Table 18 from Jónsson *et al.*^8^.						

**Table 3:** The co-occurrences of flowcell and machine type for the BM-BAM files.

	**GAIIx**	**HiSeq**	**HiSeq 2000**	**HiSeq 2500**	**HiSeq X**	**HiSeq X2**
GAIIx	8,309	0	0	0	0	0
HiSeq	0	1,115	4,946	136	0	0
HiSeq X	0	0	0	0	6,606	6,705
The rows and columns correspond to the machine and flowcell types, respectively.						

**Table 4:** The frequencies of flowcell and machine combinations for the AM-BAM files

**Machine**	**Frequency**	**Flowcell**	**Frequency**
HiSeq X	12,091	HiSeq X	6,439
GAIIx-HiSeq	1,228	HiSeq X2	5,648
HiSeq	719	GAIIx-HiSeq 2000	997
GAIIx-HiSeq-HiSeq X	473	HiSeq 2000	599
GAIIx-HiSeq X	432	GAIIx-HiSeq X2	432
GAIIx	253	GAIIx-HiSeq-HiSeq X2	271
HiSeq-HiSeq X	24	GAIIx	253
		GAIIx-HiSeq 2000-HiSeq X2	202
		GAIIx-HiSeq	139
		HiSeq 2000-HiSeq 2500	94
		GAIIx-HiSeq-HiSeq 2000	92
		HiSeq 2500	23
		HiSeq 2000-HiSeq X2	10
		HiSeq 2000-HiSeq X	8
		HiSeq-HiSeq X2	6
		HiSeq X-HiSeq X2	4
		HiSeq	2
		HiSeq-HiSeq 2000	1

**Table 5:** Summary of the variants.

**Variant type**	**Allele**	**Total**	**GATK-P**	**Phase-P**	**Imputation-P**
Indel	Biallelic	5,962,773	3,510,962	4,009,176	3,235,133
Indel	Non-biallelic	7,085,563	4,429,828	5,462,957	3,619,729
SNP	Biallelic	69,663,011	30,518,223	35,631,803	29,319,382
SNP	Non-biallelic	3,406,010	561,155	1,679,332	953,751
Total	Biallelic	75,625,784	34,029,185	39,640,979	32,554,515
Total	Non-biallelic	10,491,573	4,990,983	7,142,289	4,573,480
The columns GATK-P, Phase-P (>0.8) and Imputation-P (>0.8) correspond to number of variants passing the respective filters. Multi allelic variants were dichtomized.					

**Table 6:** Summary of the phased DNMs.

**Phased set**	**Counts**	**Fraction of the DNMs**
Three gen. imputation	15,746	0.145
Read pair tracing	31,834	0.293
Phased by both methods	4,566	0.0420
Phased by both methods, discordant (1.16%)	53	0.000487
Consensus approach	42,961	0.395
